# Trends in paediatric bloodstream infections at a South African referral hospital

**DOI:** 10.1186/s12887-015-0354-3

**Published:** 2015-04-02

**Authors:** Angela Dramowski, Mark F Cotton, Helena Rabie, Andrew Whitelaw

**Affiliations:** Department of Paediatrics and Child Health, Division of Paediatric Infectious Diseases, Faculty of Medicine and Health Sciences, Stellenbosch University, PO Box 19063, Tygerberg, 7505 South Africa; Department of Medical Microbiology, Stellenbosch University and the National Health Laboratory Service (NHLS), Cape Town, South Africa

**Keywords:** Bloodstream infection, Sepsis, Community-acquired infection, Hospital-acquired infection, Healthcare-associated infection, paediatrics, antimicrobial resistance, vaccination, HIV

## Abstract

**Background:**

The epidemiology of paediatric bloodstream infection (BSI) in Sub-Saharan Africa is poorly documented with limited data on hospital-acquired sepsis, impact of HIV infection, BSI trends and antimicrobial resistance.

**Methods:**

We retrospectively reviewed paediatric BSI (0–14 years) at Tygerberg Children’s Hospital between 1 January 2008 and 31 December 2013 (excluding neonatal wards). Laboratory and hospital data were used to determine BSI rates, blood culture contamination, pathogen profile, patient demographics, antimicrobial resistance and factors associated with mortality. Fluconazole resistant Candida species, methicillin-resistant *Staphylococcus aureus* (MRSA), multi-drug resistant *Acinetobacter baumannii* and extended-spectrum beta-lactamase (ESBL) producing *Enterobacteriaceae* were classified as antimicrobial resistant pathogens.

**Results:**

Of 17001 blood cultures over 6 years, 935 cultures isolated 979 pathogens (5.5% yield; 95% CI 5.3-5.7%). Contamination rates were high (6.6%, 95% CI 6.4-6.8%), increasing over time (p = 0.003). Discrete BSI episodes were identified (n = 864) with median patient age of 7.5 months, male predominance (57%) and 13% HIV prevalence. BSI rates declined significantly over time (4.6–3.1, overall rate 3.5 per 1000 patient days; 95% CI 3.3–3.7; Chi square for trend p = 0.02). Gram negative pathogens predominated (60% vs 33% Gram positives and 7% fungal); *Klebsiella pneumoniae (154; 17%), Staphylococcus aureus (131; 14%)* and *Escherichia coli (97; 11%)* were most prevalent. Crude BSI mortality was 20% (176/864); HIV infection, fungal, Gram negative and hospital-acquired sepsis were significantly associated with mortality on multivariate analysis. Hospital-acquired BSI was common (404/864; 47%). Overall antimicrobial resistance rates were high (70% in hospital vs 25% in community-acquired infections; p < 0.0001); hospital-acquired infection, infancy, HIV-infection and Gram negative sepsis were associated with resistance. *S. pneumoniae* BSI declined significantly over time (58/465 [12.5%] to 33/399 [8.3%]; p =0.04).

**Conclusion:**

Although BSI rates declined over time, children with BSI had high mortality and pathogens exhibited substantial antimicrobial resistance in both community and hospital-acquired infections. Blood culture sampling technique and local options for empiric antimicrobial therapy require re-evaluation.

## Background

The epidemiology of paediatric bloodstream infection (BSI) in Africa is poorly documented. A meta-analysis of prospective studies of community-acquired BSI [1] identified 22 eligible studies (four in Southern Africa) [2-5], where non-typhoidal *Salmonella*, *E. coli*, *S. aureus* and *S. pneumoniae* infection predominated. Despite published descriptions of community-acquired sepsis in African children [1-9] data on hospital-acquired BSI are extremely limited [10,11]. It is estimated that healthcare-associated BSI may be responsible for 25000 deaths in African children annually [12]. Overall, incidence rates of healthcare-associated infection in developing countries are thought to be at least double that of high-income settings [13]. Further research on the epidemiology of hospital-acquired BSI in African children is needed to quantify the burden and better understand contributory factors.

Significant changes in BSI epidemiology among African children are expected, given increasing access to HIV prevention programmes, paediatric antiretroviral therapy and inclusion of pneumococcal conjugate vaccine (PCV) in the immunisation schedule [14,15]. In light of globally increasing antimicrobial resistance [12], regional antimicrobial resistance prevalence and the efficacy of empiric antibiotic therapy for paediatric BSI in Sub-Saharan Africa also require evaluation. Descriptions of increasing prevalence of *Enterobacteriaceae* BSI isolates producing extended-spectrum beta-lactamases (ESBL) are concerning given the limited availability of appropriate antibiotics in many African countries [16].

The difficulties in obtaining paediatric blood culture specimens and the increased yield with higher volume blood inoculum are well described [8]. Of concern in our region are two recent audits [17,18] reporting low pathogen yields and high blood culture contamination rates (exceeding accepted rates of < 3%) [19]. We examined trends in paediatric BSI epidemiology over six years at a single academic institution, determining rates of bacteraemia, blood culture yield and contamination, BSI-associated mortality and prevalence of antimicrobial resistance. We also investigated the association between bacteraemia, antimicrobial resistance and HIV infection.

## Methods

### Setting

We retrospectively reviewed paediatric BSI at Tygerberg Children’s Hospital (TCH) in Cape Town, South Africa between 1 January 2008 and 31 December 2013 (excluding neonatal wards). The TCH admits sick infants and children (0–14 years) requiring general (70%) or specialised (30%) paediatric care (haematology/oncology, nephrology, gastroenterology, infectious diseases, cardiology, neurology, pulmonology, paediatric surgery, endocrinology) to one of six wards (153 beds; 85% occupancy rate in 2013). The 10-bedded medical/surgical paediatric intensive care unit (PICU) has an 89% occupancy rate (2013). Critically-ill children requiring ventilation or inotropic support are preferentially managed in the PICU but also on the wards when PICU is full.

The antenatal HIV prevalence in the Western Cape Province was 16.9% in 2012 (versus 29.5% nationally) [20]. Among children aged 2–14 years, HIV prevalence in the Western Cape Province was 0.7% in 2012 (versus 2.4% nationally) [21]. Antiretroviral therapy has been available since 2004, including prevention of mother-to-child HIV infection transmission, with transmission rates of 3.9% in 2010 [22]. HIV testing with informed consent is performed if the child’s status is unknown, using an HIV PCR if < 18 months and HIV Elisa if > 18 months. If the HIV status is already known, no laboratory sample is submitted. Thus for this study only HIV tests taken at the time of hospitalisation, or taken at prior hospital visits were accessible.

Immunisation coverage in the Cape Metropolitan area in 2011/12 was 87.5% among the population < 1 year of age (including BCG, polio, diphtheria/tetanus/pertussis, hepatitis B and measles vaccines; rotavirus was introduced in 2009) [23]. *Haemophilus influenzae* serotype B (Hib) conjugate vaccine was introduced in 1995 and PCV for *S. pneumoniae* in 2009 (7-valent) and 2011 (13- valent) [15].

### Investigation and management of BSI

Blood cultures are obtained from all children with suspected sepsis or severe infection with a focal site (e.g. pneumonia, cellulitis). Empiric antibiotic therapy for community-acquired sepsis depends on the presumed site of infection, but usually includes either ceftriaxone or ampicillin and gentamicin. Empiric treatment of hospital-acquired infection usually includes meropenem, or ertapenem if *Pseudomonas aeruginosa* is considered unlikely and meningitis is excluded. Vancomycin is added if MRSA is considered a likely pathogen e.g. with suspected central line sepsis or soft tissue infection in hospital.

### Blood culture sampling and analysis

A single blood culture sample (one bottle) is submitted from most patients, unless infective endocarditis is suspected. Local guidelines recommend inoculation of at least 2 mL of blood into paediatric blood culture bottles, however for older children, larger blood inoculums of 5 – 10 ml are encouraged. Blood cultures are taken at the discretion of attending clinicians and transferred to the on-site National Health Laboratory Service (NHLS) microbiology laboratory for processing in an automated system. Prior to April 2011 the Bactec system (Becton Dickinson, New Jersey, United States) was used; and thereafter the BacT/Alert system (BioMerieux, Marcy l′Etoile, France), in line with NHLS policy. For both systems, paediatric-specific culture bottles were used (Becton Dickinson BACTEC Peds Plus/F and thereafter BacT/ALERT® PF bottles). Both contain specialized media that accommodate small-volume samples (≤3 mL of blood) and resin for antibiotic neutralization. If bacterial growth is detected in the bottles, a Gram stain is performed, the sample sub-cultured onto appropriate media based on the Gram stain and incubated overnight. Further identification and antimicrobial susceptibility testing of clinically significant isolates is performed with the automated Vitek II system (BioMerieux, Marcy l’Etoile, France), using annually published Clinical and Laboratory Standards Institute (CLSI) breakpoints [24]. Pneumococcal isolates were considered non-susceptible to penicillin at minimum inhibitory concentrations (MICs) ≥0.12 mg/L using the meningitis breakpoints for parenteral penicillin [24].

### Data analysis

All positive blood cultures from the paediatric wards over the six year study period were extracted from the computerised laboratory database; demographic data was obtained from the laboratory and hospital admissions database. BSI rates, blood culture contamination, pathogen profile, patient demographics and factors associated with antimicrobial resistance and BSI mortality were determined. Organisms were categorised using the United States Centers for Disease Control (US CDC) list of pathogens and contaminants; common commensals defined by the CDC include diphtheroids [Corynebacterium spp. not C. diphtheriae], Bacillus spp. [not B. anthracis], Propionibacterium spp., coagulase-negative staphylococci [including S. epidermidis], viridans group streptococci, Aerococcus spp., and Micrococcus spp [25]. Positive blood cultures obtained < 72 hours after admission were classified as community-acquired sepsis. Those obtained >72 hours after admission were considered hospital-acquired sepsis. All positive blood cultures isolating the same pathogen within 14 days were considered a single episode of BSI. Fluconazole resistant Candida species, methicillin-resistant *Staphylococcus aureus* (MRSA), multi-drug resistant *Acinetobacter baumannii* (resistant to at least 3 classes of antimicrobials) and extended spectrum B-lactamase (ESBL)-producing *Enterobacteriaceae* were classified as antimicrobial resistant pathogens using proposed standard definitions [26].

### Statistical analysis

The BSI rate was calculated by dividing the total number of BSI episodes by the total inpatient days accumulated during the 6 year period. The pathogen and contamination rates were calculated by dividing the number of blood cultures yielding pathogens and contaminants respectively by the total number of blood culture requested. Continuous and categorical variables were compared using student t tests and Chi square analysis respectively. A Chi square test for linear trend was used to assess change in rates over the study period. To determine factors associated with mortality from BSI and antimicrobial resistance, binary logistic regression analyses were performed. A p-value below 0.05 was considered statistically significant. Stata Statistical Software version 13.0 IC (College Station, TX: StataCorp LP) was used.

### Ethical approval

Ethical approval and waiver of individual informed consent was obtained from the Human Health Research Ethics committee of Stellenbosch University (S13/09/171).

## Results

**Figure 1:**
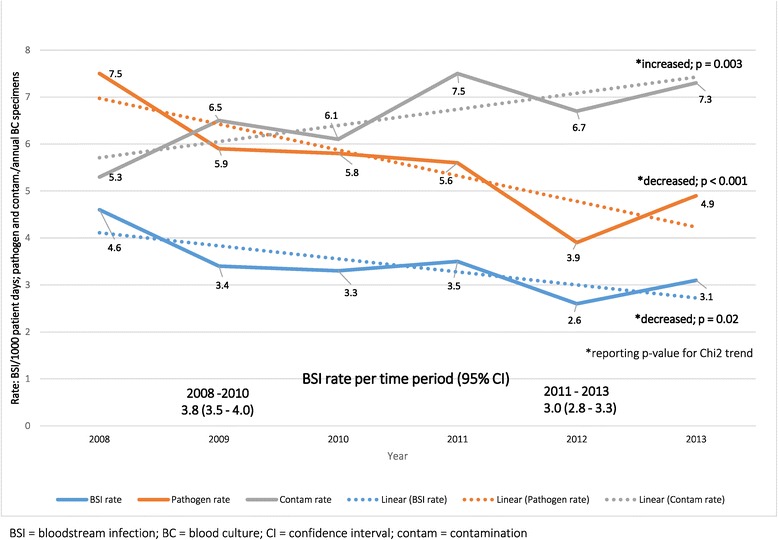
**Trends in bloodstream infection, pathogen and contamination rates (2008–2013).** BSI rates (blue) declined significantly (from 4.6 to 3.1 per 1000 patient days; Chi square for trend p = 0.02). Blood culture contamination rates (grey) were high (1123/17001 [6.6%]; 95% CI 6.4-6.8%) exceeding pathogen yield (orange) and increased over time (p = 0.003).

For 63209 children hospitalized over the study period, 17001 blood culture specimens were submitted; 1 blood culture per 3.7 admissions or 68.6 specimens/1000 patient days. From 935 culture–positive specimens, 979 BSI pathogens were isolated (5.5% yield; 95% CI 5.3-5.7%). Blood culture contamination rates were high (1123 contaminated blood cultures from 17001 blood culture specimens submitted [6.6%]; 95% CI 6.4-6.8%), increasing over time (p = 0.003) (Figure 1). Coagulase-negative staphylococci (CoNS) were the most commonly isolated contaminant (650/1123; 57.9%), followed by non-pathogenic streptococci (75/1123; 6.7%), Bacillus species (74/1123; 6.6%), Micrococcus species (63/1123; 5.6%) and diphtheroids (61/1123; 5.4%).

Of 864 discrete BSI episodes, 818 (94.7%) were monomicrobial infections and 46 (5.3%) were polymicrobial (42 with 2 pathogens; 4 with 3 pathogens). The overall BSI rate was 3.5 per 1000 patient days (95% CI 3.3–3.7). A significant decline in the BSI rate from 4.6 in 2008 to 3.1 in 2013 (Chi square for trend p = 0.02; Figure 1) was noted despite substantial increases in hospitalizations (7537 to 11201 [49%]) and number of blood cultures (7816 vs 9185 [17%]) submitted between 2008 and 2013.

The median age of patients with BSI episodes was 7.5 months with male predominance (57.4%) and 13.4% HIV prevalence (however the test positivity rate was 20.6% [116/564], when children of unknown HIV [n = 299] status were excluded) (Table 1). Most patients (679; 78.6%) had blood cultures submitted from general paediatric wards i.e. were not yet in the PICU. Nearly half of all BSI episodes were hospital-acquired (404; 46.8%), with a median hospital stay of 17 days (IQR = 8 – 33.5 days) before onset of BSI. The overall rate of hospital-acquired BSI was 1.63 episodes per 1000 patient days (404/247969; 95% CI 1.49 – 1.78) and declined between 2008–2010 and 2011–2013 (from 1.79 to 1.49/1000 patient days; p = 0.06). The risk of developing hospital-acquired BSI during hospitalisation was 6.4 per 1000 admissions (95% CI 5.8 – 7.0).

**Table 1:** **Demographic profile of paediatric patients with bloodstream infection**

**Variable**	**Number (n)**	**Percentage (%)**	**Age**	**Age**	**Age**	**p-value**
			**<1 yr**	**1 - 5 yrs**	**5 - 14 yrs**	
			**(n; %)**	**(n; %)**	**(n; %)**	
Laboratory confirmed-BSI episodes*	864	100%	506 (58.6)	206 (23.8)	152 (17.6)	-
**Median age (months)**	7.5	IQR	3.2	18.2	96	-
		2.9-23.8				
**Male**	496	57.4	298 (58.9)	111 (53.9)	89 (58.5)	0.41
**HIV status**						
- Positive	116	13.4	68 (13)	31 (15)	17 (11)	
- Negative	448	52	271 (54)	101 (49)	76 (50)	0.57
- Unknown	299	34.6	167 (33)	74 (36)	59 (39)	
**Onset of BSI** ^**#**^						
- Community-acquired	460	53.2	244 (53)	133 (29)	83 (18)	<0.001
- Hospital-acquired	404	46.8	262 (65)	73 (18)	69 (17)	
**Ward at BSI diagnosis**						
- Intensive care	185	21.4	140 (28)	33 (16)	12 (8)	<0.001
- General ward	679	78.6	366 (72)	173 (84)	140 (92)	
**BSI outcome**						
- Died	176	20.4	109 (22)	45 (22)	22 (14)	0.13
- Survived	688	79.6	397 (78)	161 (78)	130 (86)	
**Crude mortality rate by age**	-	20.4	21.5	21.8	14.5	-

*BSI episodes: blood culture sampling episodes that yielded a pathogen, excluding blood cultures that isolated the same organism within 14 days of the original sampling episode; IQR = interquartile range; ^#^CA-BSI = BC submitted within first 72 hours of admission; HA-BSI = BC submitted > 72 hours after admission. Continuous and categorical variables were compared using student t tests and Chi square analysis respectively; p < 0.05 was considered statistically significant.

Gram negative organisms predominated (60.2%) followed by Gram positives (32.4%) and fungi (7.4%). *Klebsiella pneumoniae* (154; 17%), *Staphylococcus aureus* (131; 14%) and *Escherichia coli* (97; 11%) were most prevalent (Table 2). The profile and proportional representation of pathogens varied markedly by location (ward vs PICU) and by place of onset. Gram positive pathogens were more prevalent in community-acquired isolates and Gram negative and fungal pathogens in hospital-acquired sepsis. The spectrum and ranking of BSI pathogens among children known to be HIV infected was similar to that of HIV uninfected patients, except for *S. pneumoniae* which was more common in HIV infected children (20/116 [17.2%] vs 30/448 [6.7%]; p < 0.001).

**Table 2:** **Microbiological profile of paediatric bloodstream infection episodes (n = 864)**

**BSI episodes**	**n**	**%**	Total pathogens isolated from 864 BSI episodes*		
Monomicrobial	818	94.7	914		
Polymicrobial					
- 2 pathogens	42	4.8			
- 3 pathogens	4	0.5			
**Gram negatives**	**Organism**	**n = 550**	**% of Gram negatives**	Organism rank*	
Enterobacteriaceae	*K. pneumoniae*	154	28%	1	
	*E. coli*	97	18%	3	
	*E. cloacae*	30	5%	7	
	*S. marcescens*	19	3%	10	
	*Salmonella* spp	18	3%		
	(non-typhi)				
	Klebsiella spp	12	2%		
	Other (9 different genera)	30	5%		
	Total	360	65%		
Non-fermenting Gram negative bacilli	*A. baumannii*	78	14%	5	
	*P. aeruginosa*	20	4%	9	
	*Acinetobacter* spp	16	3%		
	*S. paucimobilis*	9	2%		
	Other (9 different genera)	20	4%		
	Total	143	26%		
Other Gram negative organisms	*H. influenzae*	23	4%	8	
	*N. meningitidis*	11	2%		
	Other (6 different genera)	13	3%		
	Total	47	9%		
**Gram positives**	**Organism**	**n = 296**	**% of Gram positives**	Organism rank*	
Staphlylococci	*Staphylococcus aureus*	131	44%	2	
Streptococci	*Streptococcus pneumoniae*	91	31%	4	
	Group B *Streptococcus*	19	6%	10	
Enterococci	*Enterococcus* spp	46	16%	6	
Other Gram positive organisms	Other (4 different genera)	9	3%		
**Fungi**	**Organism**	**n = 68**	**% of Fungi**	Organism rank*	
**Candida spp**	*Candida albicans*	21	31%	9	
	*Candida tropicalis*	12	18%		
	*Candida parapsilosis*	9	13%		
	*Candida glabrata*	3	4%		
	*Candida krusei*	2	3%		
	All other *candida* spp	18	27%		
**Other fungi**	*Aspergillus* spp,	1	4%		
	*Trichosporon* spp,	1			
	unidentified yeast	1			
**BSI pathogens (n = 914) by type and place of infection onset for 864 BSI episodes**					
**Community-acquired (CA-BSI) pathogens**			**Hospital-acquired (HA-BSI) pathogens**		
**n = 477**			**n = 437**		
**CA-BSI ward pathogens** ^**#**^	n = 433	%	**HA-BSI ward pathogens** ^**#**^	n = 275	%
*- Staphylococcus aureus*	90	21	*- Klebsiella pneumoniae*	86	31
- *Streptococcus pneumoniae*	81	19	*- Candida spp*	28	10
- *Escherichia coli*	67	15	*- Acinetobacter baumanni*	26	10
- Other	195	45	*- Other*	135	49
**CA-BSI ICU pathogens** ^**#**^	n = 44	%	**HA-BSI ICU pathogens** ^**#**^	n = 162	%
*- Escherichia coli*	9	21	*- Acinetobacter baumanni*	42	26
*- Klebsiella pneumoniae*	7	16	*- Klebsiella pneumoniae*	33	20
- *Staphylococcus aureus*	5	11	*-* Candida spp	22	14
- Other	23	52	- Other	65	40

*Total pathogens isolated from 864 BSI episodes = 914 pathogens (818 monomicrobial + polymicrobial 42 × 2 isolates + 4 × 3 isolates) ^#^CA-BSI = BC submitted within first 72 hours of admission; HA-BSI = BC submitted > 72 hours after admission; ICU = intensive care unit; *Organism rank reported for the top ten isolates only.

While *H. influenzae* BSI episodes increased between 2008–2010 and 2011–2013 (7/465 [1.5%] to 15/399 [3.8%]; p = 0.05), the proportion of *H. influenzae* serotype B isolates remained similar (3/7 [42.9%] vs 5/15 [33.3%]; p =0.65). However, *S. pneumoniae* BSI declined significantly (58/465 [12.5%] to 33/399 [8.3%]) (p = 0.04). Pneumococci as a percentage of all Gram positive pathogens declined from a pre-vaccine high of 43.5% (27/62) in 2008 to 13.1% (8/61) in 2013 [p < 0.001]. The proportion of pneumococcal BSI due to vaccine-serotypes (accounting for PCV7 and PCV13) did not decrease significantly over the two time periods, despite PCV-13 giving broader coverage (20/68 [29.4%] vs 6/23 [26%], p = 0.99). The proportion of pneumococcal BSI exhibiting penicillin-resistance remained unchanged between 2008–2010 and 2011–2013, (18/58 [31%] vs 10/33 [30.3%]; p = 1.0).

Overall crude BSI mortality was 20.4% (176/864); patients with hospital-acquired BSI experienced higher mortality than community-acquired BSI (25% [101/404] vs 16.3% [75/460]; p = 0.002). The pathogen associated with the highest BSI mortality was *Acinetobacter* spp (p = 0.03) at 38% (30/78), followed by *Candida* species (31%; 20/65) and *E coli* BSI (24%; 23/97). HIV, fungi, Gram negative organisms and hospital-acquired sepsis were significantly associated with BSI mortality on multivariate analysis (Table 3).

**Table 3:** **Bloodstream infection-associated mortality**

**BSI-associated mortality**	**Number (n)**	**Percentage (%)**	**p-value**	
**Total BSI-associated deaths**	176	100	**-**	
**Male**	97	55.1	0.49	
**Median age (months) IQR**	7.2	IQR 3–14.7	**-**	
**HIV status**				
- Positive	34	19.3		
- Negative	87	49.4	0.03	
- Unknown	55	31.3		
**Onset of BSI** ^**#**^				
- Community-acquired	75/460	16.3	0.002	
- Hospital-acquired	101/404	25		
**Factors associated with mortality from BSI**				
**Variable assessed**	**Univariate analysis (p-value)**	**Multivariate analysis (p-value)**	**Odds ratio**	**95% CI**
Length of stay prior to BSI onset	<0.001	0.11	-	**-**
Age category	0.13	0.44	-	-
Gender	0.49	0.32	-	-
HIV status (positive)	0.03	0.02	1.74	1.1 - 2.8
Year of BSI	0.89	0.78	-	-
Place of BSI onset (hospital-acquired)	0.002	0.04	1.43	1.1 - 2.0
Type of BSI pathogen	0.001	0.03		
- Fungal			2.10	1.1 – 4.2
- Gram negative			1.88	1.2 – 2.9
Mono- vs poly-microbial BSI	0.72	0.6	-	-
ICU vs general ward at BSI onset	<0.001	0.001	2.93	1.9 – 4.4
Antimicrobial resistance	0.06	0.83	-	-

BSI = bloodstream infection; ICU = intensive care unit; ^#^CA-BSI = BC submitted within first 72 hours of admission; HA-BSI = BC submitted > 72 hours after admission. To determine factors associated with mortality from BSI and antimicrobial resistance, binary logistic regression analyses were performed. A p-value below 0.05 was considered statistically significant.

**Figure 2:**
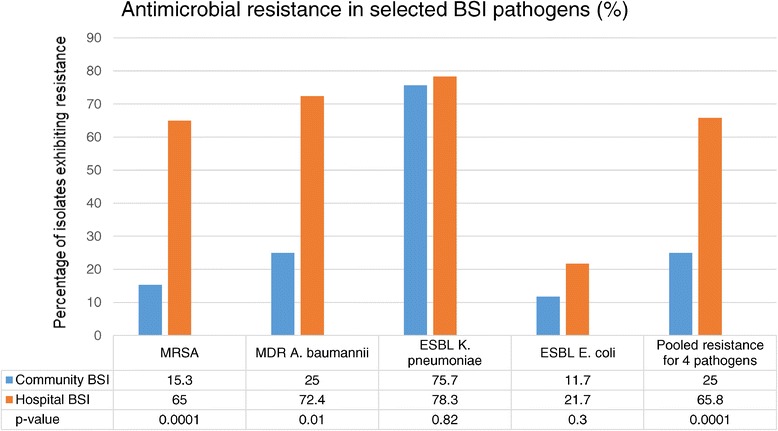
**Antimicrobial resistance (%) in selected paediatric bloodstream infection pathogens.** Methicillin-resistant *Staphylococcus aureus* (MRSA), multi-drug resistant *Acinetobacter baumannii* (resistant to at least 3 classes of antimicrobials) and extended spectrum B-lactamase (ESBL)-producing *Enterobacteriaceae* were classified as antimicrobial resistant pathogens using proposed definitions for resistance [26]. Community- vs hospital-acquired blood culture isolates of these pathogens were compared for frequency of antimicrobial resistance, individually and in a combined analysis. BSI = bloodstream infection; MDR = multi-drug resistant (according to published criteria) [24]; MRSA = methicillin resistant *Staphylococcus aureus*; ESBL = extended spectrum beta-lactamase producer; Community BSI = community-acquired BSI; Hospital BSI = hospital-acquired BSI; Pooled resistance for four bacterial pathogens = MRSA, MDR *A. baumanni*, ESBL *K. pneumoniae* and ESBL *E.coli*.

Prevalence of antimicrobial resistance was assessed among a subset of pathogens, focussing on fluconazole resistant Candida species and four bacterial pathogens: MRSA, multi-drug resistant *Acinetobacter baumannii* and ESBL-producing *E coli* and *K. pneumoniae* (Figure 2). No carbapenem resistant Enterobacteriaceae (CRE) or vancomycin resistant Enterococci (VRE) were isolated. For the four selected bacterial pathogens, antimicrobial resistance prevalence among community isolates was 25% compared with 70% among hospital-acquired isolates (p < 0.001). For *A. baumannii* and MRSA, resistance rates were significantly higher among hospital-acquired isolates, whereas *E. coli* and *K. pneumoniae* isolates had similar prevalence of ESBLs among hospital- and community-acquired isolates. Among the 65 Candida species, all 21 *C. albicans* isolates were fluconazole susceptible, while 22 of the 44 (50%) non-albicans Candida species were fluconazole resistant. The prevalence of antimicrobial resistance did not differ significantly between 2008–2010 and 2011–2013 for the selected pathogens [Chi square for trend p = 0.24 and p = 0.14 respectively]. Factors associated with antimicrobial resistance on multivariate analysis included hospital-acquired infection, infancy, HIV-infection and Gram negative sepsis (Table 4).

**Table 4:** **Factors associated with antimicrobial resistance**

**Variable assessed**	**Univariate analysis (p-value)**	**Multivariate analysis (p-value)**	**Odds ratio**	**95% CI**
Length of stay prior to BSI onset	<0.001	0.53	-	-
Age category (infants)	<0.001	0.003	1.92	1.2 – 3.1
Gender	0.8	0.92	-	-
HIV status (positive)	<0.001	<0.001	2.64	1.7 – 4.2
Year of BSI	0.4	0.19	-	-
Place of BSI onset (hospital-acquired)	<0.001	<0.001	3.68	2.7 – 5.1
Type of BSI pathogen				
- Gram negative	<0.001	<0.001	1.99	1.4 – 2.9
Mono- vs poly-microbial BSI	0.18	0.84	-	-
ICU vs general ward at BSI onset	<0.001	0.06	-	-

BSI = bloodstream infection; ICU = intensive care unit; Hospital-acquired BSI = BC submitted > 72 hours after admission. To determine factors associated with antimicrobial resistance, binary logistic regression analyses were performed. A p-value below 0.05 was considered statistically significant.

Susceptibility to different combinations of empiric antimicrobial therapy for hospital-acquired BSI was determined for all BSI isolates from 2012 and 2013 (Table 5). The combination of meropenem and amikacin was the most active against both ward and ICU BSI isolates, based on the *in-vitro* susceptibility test results (overall 82/133; 83.6% of isolates susceptible to one or both agents).

**Table 5:** Coverage achieved for hospital-acquired bloodstream infections with empiric antimicrobial regimens (2012–2013)*

**Antibiotic susceptibility**	**OVERALL n = 159**		**WARD n = 92**		**PICU n = 67**		**Ward vs PICU**
	**SUSCEPTIBLE**						
	**Number (n)**	**Percentage (%)**	**Number (n)**	**Percentage (%)**	**Number (n)**	**Percentage (%)**	**p-value**
**piperacillin tazobactam + amikacin**	122	76.7	71	77.2	50	74.6	0.7
**ertapenem**	110	69.1	71	77.2	40	59.7	0.02
**meropenem**	112	70.4	70	76	42	62.7	0.08
**meropenem + amikacin**	133	83.6	82	89.1	51	76	0.03
**meropenem + vancomycin**	119	74.8	73	79.3	46	68.7	0.14

PICU = paediatric intensive care unit; *only pathogens isolated in 2012 and 2013 were included in this analysis in order to determine recent antimicrobial resistance patterns.

## Discussion

In keeping with previous studies from Africa, gram negatives predominated in our study. *E. coli* and *Klebsiella* spp. were the most prevalent *Enterobacteriaceae*. Non-typhoidal salmonellae, a prominent BSI pathogen in malaria-endemic regions [27], was uncommon in our study. *S. pneumoniae* (the most common isolate in community-acquired BSI in African children [1]), was also prominent in our cohort. In addition, the profile of gram positive isolates changed significantly over time owing to reduced frequency of *S. pneumoniae* detection. The proportion of vaccine-serotype pneumococcal BSI isolates remained stable over time suggesting that the dramatic decline in pneumococcal sepsis rates is not solely attributable to vaccine. However, a study of invasive pneumococcal disease in South Africa did demonstrate a substantial reduction of 89% in disease caused by PCV7 serotypes in children <2 years old [28]. Other factors such as increasing antiretroviral uptake and decreasing HIV prevalence probably contributed. Fungi (especially hospital-acquired) and gram negatives were significantly associated with BSI mortality, as in other African studies [6,10,11].

The burden of paediatric BSI was concentrated among infants (58.6% of the cohort). Young age was not associated with mortality from BSI but was significantly associated with antimicrobial resistant pathogens. Overall BSI mortality in our cohort (20.4%) was higher than that reported for high income settings (11 - 14%) [29,30], but lower than other African settings (27 – 38%) [6,10,11]. Access to intensive care was not reported for the other African BSI studies and likely contributed to the lower case fatality in our cohort, although PICU facilities at our institution are very limited (only 1 in 5 patients was admitted in PICU at BSI diagnosis).

As previously reported [10], HIV-infected children were at increased risk for BSI-associated mortality and more likely to have antimicrobial resistant pathogens. Bacterial colonisation is a risk factor for later invasive infection with high rates of colonisation with antimicrobial resistant pathogens described among HIV-infected children in Cape Town [31]. It was not possible (given the study design) to compare the relative risk for BSI among HIV-infected versus HIV-uninfected children or the effect of antiretroviral therapy. A prospective study is underway at our institution to determine the relative risk for hospital-acquired infection (including BSI) among HIV-infected children.

Hospital-acquired BSI was common (nearly half of all BSI episodes), more prevalent among infants and significantly associated with mortality. The profile of hospital-acquired pathogens was distinct from that of community-acquired BSI with *Klebsiella*, *Acinetobacter* and *Candida* species predominating. In keeping with the only prospective study of hospital-acquired bacteraemia in African children, Acinetobacter sepsis had the highest case fatality rates in our cohort. The rate of hospital-acquired BSI in our study exceeded the rate of nosocomial bacteraemia in rural Kenya (1.63 versus 1.0 episodes per 1000 patient days), however our cohort had a substantially higher HIV prevalence (13.4% vs 2%) [11]. Unsurprisingly, hospital isolates exhibited significantly greater antimicrobial resistance. Unlike the Tanzanian cohort [10], antimicrobial resistance was not associated with BSI mortality, possibly due to our use of carbapenems for empiric treatment of hospital-acquired sepsis.

A worrying observation is the high rate of antimicrobial resistance among community-acquired pathogens, especially. *E.coli* and *Klebsiella* spp. Inappropriate empiric antimicrobial therapy (due to ESBL-producing and multi-resistant pathogens) predicted death in the Tanzanian cohort (OR 12.9) [10]. It is possible that pre-hospital antibiotic administration in our cohort may have falsely elevated antimicrobial resistance rates for community-acquired pathogens, by decreasing the frequency of isolation of susceptible pathogens. However our data are in keeping with pooled laboratory data (2010–2012) from public sector hospitals in South Africa demonstrating ESBL-carriage in 68% of 2774 *K. pneumoniae* BSI isolates [32]. Ongoing surveillance of antimicrobial resistance in community BSI (and monitoring of clinical outcomes among children given ineffective antibiotic therapy) is needed to determine if antibiotic guidelines need revision.

Among hospital-acquired BSI in the last two years of the study period, isolates exhibited highest susceptibility (83.6%) to meropenem plus amikacin, not currently our recommended empiric regimen. A prospective review, which includes clinical data on response to therapy is urgently needed to inform our guidelines for treatment of hospital-acquired sepsis. However, given the need for antimicrobial stewardship (and restriction of carbapenem use), it is also important to assess the efficacy of narrower-spectrum regimens (such as piperacillin-tazobactam and amikacin).

Our review of temporal trends in paediatric BSI epidemiology identified targets for quality improvement. Although not unique to our setting, blood culture contamination rates were high (double the international norm [19]) and increased over time (exceeding the rate of pathogen isolation). The annual pathogen yield declined significantly over time and in 2013 (4.9%; 95% CI 3.9 – 5.2) was substantially lower than blood culture yields from a systematic review in Africa (8.2%; 95% CI 7.9 – 8.4) [1]. There are several explanations for these findings including poor aseptic technique during specimen collection with failure to isolate pathogens because of overgrowth by c ontaminants, low sensitivity of paediatric blood cultures and sub-optimal blood volumes from children [8]. Prior administration of antibiotics also contributes to low blood culture yield: local management guidelines [33] recommend that critically-ill children referred in from primary care receive a single dose of intramuscular ceftriaxone prior to transfer.

Between the two study time periods, BSI rates declined significantly despite a substantial increase in hospitalization (measured by increased inpatient days) and despite an increase in actual numbers of blood culture specimens submitted. We suspect that improvements in PMTCT programmes, paediatric antiretroviral coverage and PCV introduction between 2008 and 2013 are partly responsible. However, declining pathogen yields (as described above) may also have artificially reduced BSI rates.

This study has several limitations, most importantly the possibility that some healthcare-associated BSI (re-admission within 30 days of hospital discharge) may have been misclassified as community-acquired, owing to the retrospective study design. We chose 72 hours as a more conservative cut-off for hospital-acquired bacteraemia (many authorities use 48 hours) to avoid possibly including some community bacteraemias as hospital-acquired. The time of blood culture collection and the time of developing symptoms/signs of infection (as opposed to blood culture collection) were not routinely available. The use of 72 hours as a cut-off, may have slightly underestimated the nosocomial bacteraemia rate. We were unable to evaluate the appropriateness of empiric therapy for community-acquired BSI, as the locally recommended treatment regimen depends on the child’s clinical presentation. Lack of standardized patient selection or technique for blood culture, lack of comprehensive clinical data or information on recent antibiotic use and/or hospitalisation are also limitations. Many patients had unknown HIV status, but were likely tested at a different facility.

Although BSI rates declined over time, we could not determine which factors and practices contributed to this trend. In addition, the BSI rates may have been significantly underestimated owing to pre-hospital antibiotic administration and inadequate blood volumes submitted for culture. We believe that change in the laboratory culturing system and average volume of blood inoculum are less likely explanations for the decline in BSI rate, since the blood culture contamination rate increased significantly over time. However, differences in pathogen yield between the two systems have been described, and the impact of a different blood culture system on the BSI rate cannot be completely discounted [34-36].

Given the relatively good resources and care at our institution (including an infection prevention and control service and PICU facilities), these findings may be more generalizable to better-resourced African settings. Recommendations for local practice arising from this study include urgent review of paediatric blood culture practice (emphasizing aseptic technique and adequate blood inoculum) and review of empiric antibiotic therapy for both community and hospital-acquired BSI.

## Conclusions

Children with BSI experienced high mortality, particularly for hospital-acquired infection. *S. pneumoniae* BSI declined after introduction of PCV and increasing antiretroviral coverage. Pathogens (both community- and hospital-acquired) exhibited substantial antimicrobial resistance. Although BSI rates declined, blood culture contamination rates increased; blood culture sampling technique and local options for empiric antimicrobial therapy require re-evaluation.

## Abbreviations

BSI: Bloodstream infection
CI: Confidence interval
ESBL: Extended-spectrum beta-lactamase
HIV: Human immunodeficiency virus
MRSA: Methicillin-resistant Staphylococcus aureus
PCV: Pneumococcal conjugate vaccine
PICU: Paediatric intensive care unit
PMTCT: Prevention of mother to child transmission of HIV
